# Molecular Characterization of Methicillin-Resistant* Staphylococcus aureus* Isolates, Isolated from a Burn Hospital in Southwest Iran in 2006 and 2014

**DOI:** 10.1155/2018/1423939

**Published:** 2018-05-20

**Authors:** Ehsanollah Ghaznavi-Rad, Alireza Ekrami

**Affiliations:** ^1^Molecular Research Center, Faculty of Medicine, Arak University of Medical Sciences, Arak, Iran; ^2^Department of Microbiology & Immunology, Faculty of Medicine, Arak University of Medical Sciences, Arak, Iran; ^3^Infectious and Tropical Diseases Research Center, Health Research Institute, Ahvaz Jundishapur University of Medical Sciences, Ahvaz, Iran

## Abstract

**Objectives:**

Incidence of methicillin-resistant* Staphylococcus aureus* (MRSA) is increasing every year, especially in burn patients with a high rate of morbidity and mortality. Molecular and epidemiologic studies are useful practices for understanding the relatedness of isolates in a single patient or a hospital. This study aimed at determining molecular characterizations of isolates collected in 2006 and 2014 using* S. aureus*-specific staphylococcal protein A (Spa) typing and Multilocus Sequence Typing (MLST) methods.

**Materials and Methods:**

Totally, 71 MRSA isolates were collected during the last two studies (2006 and 2014) from burn patients at Taleghani Burn Centre. After confirmation, all isolates were analysed using MLST and* Spa* typing methods.

**Results:**

We reported the emergence of Spa type t021, ST-30-IV MRSA isolates, which were PVL-positive in 14.6% of the cases and t12366, ST-8-IV isolates, which were PVL-negative in 9.8% of the cases. In 2014 study, Spa typing of MRSA isolates revealed five different spa types. Overall, in two studies, t037, ST-239, SCC*mec* III, and CC8 were predominant clones and they were reported in 63% of the cases.

**Conclusion:**

The predominance of ST-239 in this region during the last eight years is a major concern. It also has a disturbing impact on the management of staphylococcal infections. Moreover, the SCC*mec* type IV strain is able to disseminate rapidly in hospital environments, demanding an improvement in infection-control policy.

## 1. Introduction

Burn patients are more susceptible to the colonization and infection with opportunistic bacteria. Multidrug-resistant* Staphylococcus aureus, Pseudomonas aeruginosa*,* Klebsiella *spp., and various coliform bacilli are the main causes of nosocomial infections in such patients. Methicillin-resistant* Staphylococcus aureus* (MRSA) strains are the main cause of bacterial nosocomial infection in hospitals and communities worldwide [1]. MRSA infections are associated with high frequency of morbidity and mortality in immune-compromised patients, particularly burn patients [2].* mecA* gene that is responsible for resistance to methyl-penicillins including methicillin is located on a mobile staphylococcal cassette chromosome mec (SCC*mec*) elements and encodes an altered Penicillin Binding Protein (PBP2a), with low affinity for all *β*-lactam antibiotics even in the same SCC*mec* type. The SCC*mec* regions may be variable and thus have been considered as targets for subtyping SCC*mec* elements in epidemiological studies [3].

Today, epidemiologic studies are used to understand the relatedness of isolates that are causing an infection in one patient or the entire hospital. These techniques determine whether the isolates collected from different environments are of the same or different strains, as well as an infection relapse or reinfection. At present, genotypic methods for epidemiological investigation of MRSA include* Spa* typing, MLST, and Pulsed Field Gel Electrophoresis (PFGE) [4].

The* Spa *typing method involves sequencing of the polymorphic X region or the short sequence repeat region of the protein A gene. This method is appropriate for discrimination in outbreak investigation. The data required for* Spa *typing are gathered from a single locus, unlike MLST, in which data from a number of loci are combined to represent housekeeping genes for typing of* S. aureus *by PCR [5].

Numerous molecular characterization and epidemiological studies have indicated that MRSA frequency throughout Iran has been increasing, especially in the last decade [6]. However, regionally, the rates differ dramatically. According to previous studies, a high frequency of MRSA has been reported in Taleghani Burn Hospital, the only burn reference centre in Ahvaz, southwest of Iran [7, 8]. Thus, considering the significance of following up and monitoring MRSA isolates among burn patients, the current study was focused on determining the molecular characterization of isolates using* Spa *typing and MLST methods. The isolates that were included in this study were collected in 2006 and 2014 in two separate studies and the main object was to monitor the genotypic variation at two different time periods in this burn hospital.

## 2. Materials and Methods

### 2.1. Location and Sampling Procedures

Taleghani Burn Centre is a 160-bed teaching referral hospital. It has approximately 1200 patients with all stages of burns admitted annually. This hospital is the main burn centre in Ahvaz City, the capital of Khuzestan, the largest province in southwest Iran. More than 30% of the patients admitted to this hospital were referred from three neighbour provinces in the southwest of Iran.

Sample collection was carried out in Taleghani Burn Hospital following two studies in 2006 and 2014. A total of 71 MRSA isolates were included in this study (30 isolates from the 2006 study and 41 from the 2014 study). All staphylococci isolates have been identified by conventional microbiology and differential tests including catalase production, coagulase activity, clumping factor, DNase production, blood agar hemolysis, and susceptibility to novobiocin (5 *μ*g). MRSA isolates were identified through a disk diffusion method by using a cefoxitin 30 *μ*g disk (Mast, UK) considering the Clinical and Laboratory Standards Institute (CLSI-2015). All the MRSA strains were evaluated for resistance to Vancomycin by *E*-test (bioMérieux) [9].

### 2.2. Molecular Characterization

Phenotypic identification of all the* S. aureus* isolates was confirmed using the SA442 primer, which is specific for* S. aureus*. In addition, an already established PCR assay was used for the detection of* mecA* gene [10]. All the isolates were assessed for the presence of resistance genes* VanA* and* VanB*, as well as PVL cytotoxin gene by specific primers using a PCR-based method. For amplification of* Spa* gene, the short sequence repeats (SSR) of the X region were amplified with the following primers: forward: 5′-AGACGATCCTTCGGTGAGC-3′ and reverse: 5′-GCTTTTGCAATGTCATTTACTG-3′, as described by Shopsin et al. [11]. PCR amplification was performed according to the procedure described by Harmsen et al. (2003): initial denaturation of 10 min at 95°C, followed by 30 cycles of denaturation for 30 seconds at 95°C, 30 seconds of annealing at 60°C, and 45 seconds of extension at 72°C and a final extension at 72°C for 10 min.* S. aureus* ATCC 25923 contains* Spa* gene considered as positive control. SCC*mec* typing was carried out using established specific primers for amplification of* mec*,* ccr*, and *J* region of staphylococcal cassette chromosome* mec* as described by Ghaznavi-Rad et al. [10].

MLST was carried out as described by Enright et al. In this assay, seven standard housekeeping genes* (arcC, aroE, glpF, gmk, pta, tpi, *and* yqiL)* were amplified in the test samples using the corresponding primers. PCR condition for amplification of all seven genes has been described on the MLST website (http://saureus.mlst.net). The entire PCR products were purified and sequenced (Bioneer Co., Korea). The gene sequences were recorded on the MLST website (http://saureus.mlst.net), with defined allelic profiles and assignment to sequence type. Statistical analyses were determined using SPSS for Windows statistical software (version 18; SPSS, Inc., Chicago, IL, USA).

## 3. Result

From January to July 2006 and from January to July 2014, 30 out of 50 (60%) isolates and 41 (out of 60, 68.3%)* S. aureus* isolates, respectively, causing infections in the burn hospital were identified as MRSA by standard tests. There was no significant difference between the frequencies of MRSA isolates in two studies (*P* = 0.065). MRSA isolates were isolated from wounds (25, 61%) and blood (16, 39%) samples. All the strains were positive for* Sa442* and* mecA* gene as identification marker and gene encoding resistance to methicillin. All the isolates were susceptible to Vancomycin according to *E*-test method and no Vancomycin-resistant genes were found using PCR assay.

Overall, in the 2014 study, 22 (53.6%) of MRSA strains were isolated from female patients and statistical analysis found no significant association between MRSA infection and gender.

All the MRSA isolates were isolated from the specimens obtained 72 hours after admission to the hospital.

Molecular characterization of hospital-acquired MRSA strains has been shown in Table 1. In 2006 study, 30 isolates (100%) belonged to Spa type t037, ST-239, SCC*mec* III, and CC8 and all were PVL-negative. Meanwhile, Spa typing of 41 MRSA isolates in 2014 revealed five different Spa types. In 2014, although t037, ST-239, and SCC*mec* III are still the predominant clones, 15 (36.6%), four other Spa types have emerged. Among them, t631, ST-239, and SCC*mec* III were PVL-negative, 10 (24.4%), and t690 SCC*mec* IV which belongs to ST-88 was PVL-positive, 6 (14.6%). An interesting finding of this study was the emergence of Spa type t021, ST-30, and SCC*mec* IV which were PVL-positive, 6 (14.6%), and t12366 and ST-8-IV which were PVL-negative, 4 (9.8%).

## 4. Discussion

This report shows the detailed genetic characteristics of MRSA isolated in a tertiary burn hospital between 2006 and 2014. Although the frequency of MRSA is high during these eight years, no changes were observed in the MRSA population structure of burn patients in our hospital during this time. About 30% of the world's population (and 21% of the Iranian population) is found to carry* S. aureus *at any time; among healthcare workers, the carriage rate is much higher and usually occurs in conjunction with MRSA [12, 13]. Many epidemiologic studies have focused on the high rate of MSRA connected to healthcare-associated (HA) infections around the globe. Each geographical region has its own endemic and epidemic strains. The result of the present study found that t037, ST-239-III has remained as the predominant clone from 2006 to 2014. Currently, we know that most nosocomial infections due to MRSA are caused by a small number of MRSA clones distributed throughout the world [14]. Results of a survey reviewing the genomic background of MRSA strains collected from 12 different Asian countries indicated that all isolated Korean and Japanese strains were clonal complex 5 (CC5), while the rest of the Asian countries were dominated by ST-239 [15]. In Iran, ST-239 was reported for the first time by Fatholahzadehin (2009) and during recent years has continued to be the main cause of MRSA infections in different parts of Iran [16].

At present, two MRSA genotypes are dominant in the isolates from Asian countries. These genotypes are distributed in two different geographic regions, Korea and Japan, but not in other Asian countries. Based on MLST analysis, CC5 has been found as a predominant clone in isolates from Korea and Japan, while MRSA from other Asian nations, such as China, India, Indonesia, the Philippines, Saudi Arabia, Singapore, Sri Lanka, Taiwan, Thailand, and Vietnam, were ST-239, except in a few cases [17]. The SCC*mec* types of MRSA clones were also distributed in the same geographical manner. Most MRSA from Eastern and Middle Eastern countries contain SCC*mec* type III or IIIA, although Parhizgari et al. (2016) have reported that, among MRSA isolated from a multicenter study in the southwest of Iran, t037, ST-239-III is the predominant clone (42.5%) [18]. Recently, there have been some reports showing the replacement of ST239 with ST22 (EMRSA-168 15) in Asian countries such as Iran, India, Kuwait, Singapore, and Malaysia [19, 20]. Emergence of ST22 is known to be the major MRSA clone in European countries, Australia, New Zealand, and Singapore and now is emerging in Asian countries, thus indicating the pandemic nature of this clone. Therefore, it can be expected that it will spread to other Asian countries within a short time.

No Vancomycin-resistant MRSA (VRSA) or intermediate MRSA was found in the present study. The first VRSA strain was reported from Tehran, Iran, in 2013 by Aligholi et al. [21]. However, VRSA was distributed in most parts of Iran by 2015 [22]. This finding suggests that Vancomycin can still be used for MRSA infection treatment in this geographical region.

Here we reported the emergence of t021-, ST30-, SCC*mec* IV-, and PVL-positive MRSA strains, which had not yet been reported in this region or in other similar studies in Iran [23]. Recently, Moghadam et al. reported MRSA isolates from the wound discharges of hospitalized patients in central Iran to be t021-, ST30-, SCC*mec* IV-, and PVL-positive in 8.9% of the cases [24]. The multilocus ST30, PVL-positive CA-MRSA strain has been found to be a major cause of outbreaks worldwide. Particularly, ST30-IV was detected in the South West Pacific, Europe, and USA but is known as the South West Pacific clone. In Asia, the ST30, PVL-positive strain was responsible for community and hospital infections in Japan and China between 1985 and 1990, respectively [25, 26]. In addition, this strain was the minor causative agent of hospital-acquired infections in Singapore and Malaysia [27]. The extensive dissemination of this clone in 188 different parts of the world is the reason why it is considered a pandemic clone [28].

In comparison to other SCC*mec* elements, SCC*mec* IV is small (21–25 kb) and more variable, which has possibly enabled it to spread easily within* S. aureus*.

Another novel finding of this study was the detection of t690, ST88, and SCC*mec* IV MRSA isolates that were PVL-negative in 14.6% of the cases. MRSA ST88 has been known as a CA-MRSA clone in some European countries, such as Portugal and Sweden, and in African countries, such as Nigeria [29, 30]. Recently, this strain has spread to many other countries in different parts of the world (e.g., Ghana and Algeria), has caused nosocomial infections, and is considered HA-MRSA [31, 32]. In the Persian Gulf region, HA-MRSA ST88 IV PVL-negative isolates have been detected in Qatari hospitals since 2013 [33]. Between 1992 and 2010, a comprehensive study in a Kuwait hospital found the ST88 IV PVL-negative clone to be a continuous strain circulating in the hospitals as HA-MRSA [34]. As these two countries are our neighbors and due to highly transmissible property of the type IV SCC*mec*, there is a possibility that this strain has entered our hospitals from these countries.

Finally, a minor clone (4, 98%) of t12366 ST8 PVL-negative was found in the present study. ST8-MSSA (methicillin susceptible* S. aureus*), which belongs to CC8, is the most likely ancestor of the first MRSA strain ST250-MRSA-I. This is because the only difference is the point mutation in the* yqiL* locus which discriminates the ST8 from ST250 strain. ST8 MRSA is one of the major causes of human infections worldwide and has gradually obtained SCC*mec* types I, II, and IV [35]. ST8 MRSA has been found as a unique clone and is responsible for community infections in Japan and Bangladesh [36, 37]. Nevertheless, ST8/SCC*mec*/IV/PVL-positive, known as USA 300, is a major cause of soft tissue infection in USA. Recently, ST8/SCC*mec*/IV, which is distinct from USA 300, has been frequently isolated from pneumonia, bacteremia, and wound and soft tissue infections [38]. The emergence of this sequence type in a burn hospital needs more attention, particularly from an infection-control committee. It can be concluded that the predominance of ST-239 during the last years in this burn hospital has been a major concern and needs special attention.

The ability of MRSA to colonize the host and induce infection and its capacity to acquire and exchange different genetic information in different clones are elements that contribute to its success as a pathogen. For instance, the penetration of ST30/ST/88/ST8 strains in our hospital has had an alarming impact on the management of staphylococcal infections. Improved professional practices and higher infection-control precautions are required for controlling and preventing the healthcare-associated infections caused by MRSA in a hospital setting. Moreover, a combination of MLST and Spa typing will allow us to more effectively monitor and recognize changes in MRSA epidemiology over time.

## Figures and Tables

**Table 1:** Molecular characterization of MRSA strains isolated in the years 2006 and 2014.

Number (%)	Spa type	MLST	CC	SCC*mec*	PVL	Year
30 (100%)	t037	ST239	8	III	Negative	2006
15 (36.6%)	t037	ST239	8	III	Negative	2014
10 (24.4%)	t631	ST239	8	III	Negative	2014
6 (14.6%)	t021	ST30	30	IV	Positive	2014
6 (14.6%)	t690	ST88	88	IV	Negative	2014
4 (9.8%)	t12366	ST8	8	IV	Negative	2014
